# Extraocular muscle regeneration in zebrafish requires late signals from Insulin-like growth factors

**DOI:** 10.1371/journal.pone.0192214

**Published:** 2018-02-07

**Authors:** Alfonso Saera-Vila, Ke’ale W. Louie, Cuilee Sha, Ryan M. Kelly, Phillip E. Kish, Alon Kahana

**Affiliations:** 1 Department of Ophthalmology and Visual Sciences, Kellogg Eye Center, University of Michigan, Ann Arbor, Michigan, United States of America; 2 Department of Biologic and Materials Sciences, School of Dentistry, University of Michigan, Ann Arbor, Michigan, United States of America; University of Minnesota Medical Center, UNITED STATES

## Abstract

Insulin-like growth factors (Igfs) are key regulators of key biological processes such as embryonic development, growth, and tissue repair and regeneration. The role of Igf in myogenesis is well documented and, in zebrafish, promotes fin and heart regeneration. However, the mechanism of action of Igf in muscle repair and regeneration is not well understood. Using adult zebrafish extraocular muscle (EOM) regeneration as an experimental model, we show that Igf1 receptor blockage using either chemical inhibitors (BMS754807 and NVP-AEW541) or translation-blocking morpholino oligonucleotides (MOs) reduced EOM regeneration. Zebrafish EOMs regeneration depends on myocyte dedifferentiation, which is driven by early epigenetic reprogramming and requires autophagy activation and cell cycle reentry. Inhibition of Igf signaling had no effect on either autophagy activation or cell proliferation, indicating that Igf signaling was not involved in the early reprogramming steps of regeneration. Instead, blocking Igf signaling produced hypercellularity of regenerating EOMs and diminished myosin expression, resulting in lack of mature differentiated muscle fibers even many days after injury, indicating that Igf was involved in late re-differentiation steps. Although it is considered the main mediator of myogenic Igf actions, Akt activation decreased in regenerating EOMs, suggesting that alternative signaling pathways mediate Igf activity in muscle regeneration. In conclusion, Igf signaling is critical for re-differentiation of reprogrammed myoblasts during late steps of zebrafish EOM regeneration, suggesting a regulatory mechanism for determining regenerated muscle size and timing of differentiation, and a potential target for regenerative therapy.

## Introduction

Loss of skeletal muscle mass, whether from degenerative disease, muscular dystrophy, denervation or trauma, is a major cause of morbidity and one of the top public health burdens [1]. In mammals, muscle injury leads to satellite cell activation and repair of focal injury, but *de novo* regeneration is not observed [2–4]. Degenerative muscle conditions result in atrophy, fibrosis and loss of muscle function [5]. Whether loss of muscle function is the result of severe muscle injury or degeneration, recovery of muscle function would require replacement of lost muscle tissue, i.e. de novo regeneration.

Our lab has discovered that in adult zebrafish, extraocular muscles (EOMs)–a subtype of skeletal muscle–can undergo de novo regeneration that is driven by myocyte reprogramming and dedifferentiation [6]. We have further characterized the early steps of EOM reprogramming, revealing important roles for epigenetic alterations, FGF signaling and autophagy in regulating proliferation by reprogrammed myoblasts [7–9].

In this work, we investigated the role of Igf signaling in zebrafish EOM regeneration. Igf family members are growth factors that play important roles in zebrafish fin [10] and heart [11] regeneration, myogenesis and muscle repair (particularly in birds and mammals) [12–16], and autophagy regulation [17–20]. Igf and Igf receptors are expressed in EOMs [21, 22] suggesting a role in EOM plasticity and force regulation. In fact, single IGF injection or sustained administration in rabbit [23], chicken [24, 25] and non-human primates [26] increased both EOM mass and force. Therefore, we hypothesized that it might also be important in EOM regeneration, and particularly in early reprogramming events.

Our data reveal that both pharmacologic and genetic inhibition of Igf signaling disrupt EOM regeneration. Interestingly, neither cell proliferation nor autophagy activation were affected by Igf signaling inhibition, indicating that it does not regulate myocyte reprogramming. Instead, histologic analysis and myosin staining of regenerating muscles indicate that Igf promotes later events in the regeneration process, i.e. myoblast terminal differentiation and fusion. We also discovered that the Akt pathway is not the target of Igf signaling in this process. We conclude that Igf signaling has an evolutionarily conserved role in the response of skeletal muscles to devastating injury, and that in zebrafish EOMs, Igf signaling is important for re-differentiation of the regenerating muscle.

## Materials and methods

### Zebrafish (*Danio rerio)* rearing and surgeries

All animal work was performed in compliance with the ARVO Statement for the Use of Animals in Ophthalmic and Vision Research, and approved by the University of Michigan Committee on the Use and Care of Animals, protocol 06034. Sexually mature adult (4–18 month old) zebrafish were spawned in our fish facility and raised per standard protocol at 28°C with a 14-h light/10-h dark alternating cycle.

Adult zebrafish were anesthetized (0.05% Tricaine Methanosulfate) and approximately 50% of the right lateral rectus (LR) muscle was surgically excised, i.e. myectomy, as described [6]. The contralateral side (left side) was used as internal uninjured control. The remaining muscle following surgery (48.42% ± 4.9%, average ± S.D.) is represented in the graph figures as a gray area (i.e. a baseline for regeneration). Fish were euthanized using anesthesia overdose followed by decapitation, and the length of the regenerating muscle was quantified by craniectomy as described previously [6]. Regeneration is represented as the relative size of the injured (right) LR muscle normalized to the length of the uninjured (left) LR control muscle (representing 100%—immediately following myectomy, the ratio is approximately 50%). All experiments were performed using 5 fish per experimental group and/or time point, unless stated otherwise in the text and/or figure legend.

### Drug treatments

BMS754807 (ChemieTek, Indianapolis, IN), an Igf1r kinase inhibitor [27], was dissolved in DMSO as a 10 mM stock and added to fish water to a final concentration of 5μM. To confirm the specificity of IGF signaling blockade, the unrelated inhibitor NVP-AEW541 (MedChem Express, Princeton, NJ), another structurally distinct Igf1r inhibitor [28], was dissolved in DMSO as a 10 mM stock and added to fish water to a final concentration of 5 μM. Up to 5 fish were treated in 1 liter of water, tanks were maintained at 28.5°C, and drug solutions were replaced every 24 h. Drug treatments were initiated immediately after surgery and no mortality was observed.

### Morpholino oligonucleotide injection and electroporation

Microinjection of morpholino oligonucleotides (MOs; Gene-Tools, LLC, Philomath, OR), a widely used technique to perform knockdown experiments in adult zebrafish [29–31], was used. Briefly, lissamine-tagged MOs were directly microinjected into the right LR muscle followed by square-wave electroporation (6 to 10 pulses at 48 V/cm, BTX ECM830 electroporator; Harvard Apparatus, Holliston, MA). Microinjections were performed 3 hours prior to LR injury, and MO uptake was confirmed via lissamine fluorescence prior to myectomy. The MO sequence for *igf1ra* and *igf1rb* genes was previously validated in zebrafish [32, 33], and a standard MO targeting a human beta-globin intron mutation was used as control. Experiments were performed using 5 fish per experimental group, unless stated otherwise in the text and/or figure legend. No mortality was detected during the experimental process.

### Specimen processing

Zebrafish heads were excised and fixed in 4% paraformaldehyde (PFA) overnight at 4°C. Decalcification was performed using either Morse’s solution. Fixed and decalcified tissues were cryopreserved with 20% sucrose in PBS, embedded in OCT (Fisher Scientific), frozen and evaluated microscopically using coronal frozen sections (12 μm). Paraffin sections (5 μm) were obtained at 5 or 7 dpi and H&E stained following standard techniques as described previously [6].

### Autophagy assessment

Adult zebrafish were incubated in 500 nM LysoTracker Red DND-99 (L-7528, Thermo Fisher Scientific, Waltham, MA) for one h and then washed in fresh fish water (3 x 20 min). During the induction of autophagy the soluble form (LC3-I) is conjugated to a form (LC3-II) that accumulates in the autophagosomes [34]. Transgenic fish expressing GFP-tagged Lc3 (GFP-Lc3) [35] were used to visualize Lc3 after LR muscle myectomy.

Then fish were processed for craniectomy as described. LysoTracker Red staining and GFP-Lc3 accumulation were quantified measuring the fluorescence intensity of the area corresponding to the regenerating muscle in pictures taken from craniectomized fish with ImageJ software (http://rsbweb.nih.gov/ij/). Pictures were taken at the same time and with the same microscope settings to minimize experimental variability.

### EdU incorporation assays

Cellular proliferation was assessed by intra-peritoneal (IP) injections of 5-ethynyl-2’-deoxyuridine (EdU) and standard detection methods [36]. Fish were anesthetized and injected with EdU (20 μl, 10 mM EdU in PBS) at 23 hours post injury (hpi) and sacrificed four hours later (27 hpi). For each experiment, 4 fish per group were analyzed. The injured muscle of each fish was analyzed and EdU+ or total (DAPI) nuclei were counted from, at least, 3 nonconsecutive sections per muscle, representing approximately 1958 total nuclei (range 1273–2449) per muscle. Cell proliferation is represented as the percentage of EdU+ nuclei in the injured muscle.

### Immunolabeling

Myosin immunohistochemistry was performed as described [6]. Negative control experiments were performed in which either primary or secondary antibodies were omitted. Briefly, slides with coronal frozen sections (12 μm) were washed in PBS for 5 min and placed in blocking solution (5% goat serum in PBS + 0.2% Tween, PBST) for 30 min. Slides were incubated in a humidity chamber overnight at 4°C in primary antibody (mouse monoclonal anti-myosin heavy chain, F59, Developmental Studies Hybridoma Bank (DSHB), University of Iowa, Iowa City, Iowa) diluted to 1:50 in PBST+1% goat serum and washed again 4 times for 5 min in PBST. Then, slides were incubated in the dark with Alexafluor 647-conjugated goat anti-mouse secondary antibody (Invitrogen) diluted 1:1000 in PBST + 1% goat serum. After 3 5-min PBS washes, slides were coverslipped using ProLong Diamond Antifade Reagent with DAPI to stain the nuclei. Sections from 7 different fish per experiment were stained and imaged.

### Western blots

Western blots were performed following standard protocols. Injured or control (uninjured) LR muscles from 10 to 15 fish were pooled and homogenized in lysis buffer containing protease (cOmplete, Roche Diagnostics Corporation, Indianapolis, IN) and phosphatase (PhosSTOP, Roche Diagnostics Corporation) inhibitors. The transgenic α actin-EGFP fish were used to visualize the muscles. Samples were sonicated and centrifuged at 10,000 g for 10 min at 4°C. Supernatant was collected and protein concentration determined using the Pierce™ BCA Protein Assay Kit (Thermo Scientific, Rockford, IL) and bovine serum albumin (BSA) as standard. Equal amounts of protein (20 μg) were loaded on 7.5% SDS polyacrylamide gels covered with a 3.9% stacking polyacrylamide gel and separated at 130 V for 1 h using Mini-Protean III (Bio-Rad, Hercules, CA). Proteins were then electroblotted onto PVDF membranes (Bio-Rad) by wet transfer (Mini Trans-Blot® Cell, Bior-rad) at 100 W for 1 h. Membranes were blocked for 1 h at room temperature with 5% BSA in TBST and incubated overnight at 4°C with primary antibody diluted in blocking solution. Anti-γ-tubulin antibody (1:10000, T5326) was obtained from Sigma-Aldrich, anti-Akt (1:1000, #9272) and anti-phospho-Akt (Ser473) (1:2000, #4060) were purchased from Cell Signaling Technology. Membranes were washed in TBST and incubated with IgG-horseradish peroxidase conjugate secondary antibody (1:10000, anti-mouse #7076 and anti-rabbit #7074 from Cell Signaling Technology) at room temperature for 1 h. Detection of signal was done using WesternBright ECL HRP substrate (advansta, Menlo Park, CA) and an Azure c500 Western Blot Imaging System (azure biosystems, Dublin, CA). Densitometric quantification of the bands was done with ImageJ software (http://rsbweb.nih.gov/ij/). The intensity of the protein of interest is normalized to the intensity of the tubulin band and represented in relative units (R.U.).

### Statistics

**Fig 1:**
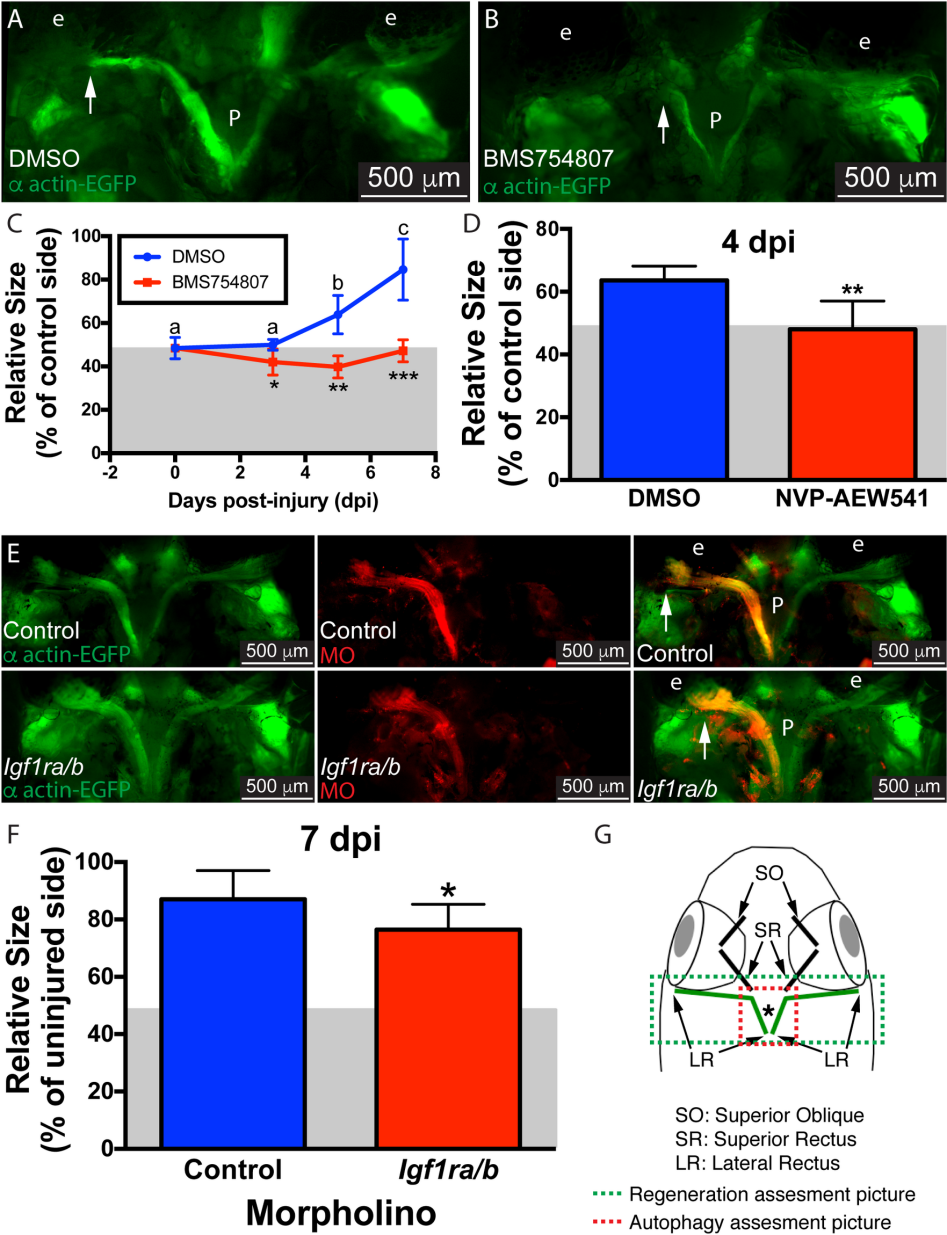
Inhibition of Igf1r impairs muscle regeneration. Myectomized α-actin-EGFP fish treated with the Igf1r inhibitor BMS754807 (B) or DMSO (A) for 5 days. At selected time points (3, 5, and 7 dpi), the length of the regenerating muscle was measured as described (C), values are averages ± SD (n = 5–6). For each group (DMSO or BMS754807), differences among time points were analyzed by ANOVA. Different letters (lower case over DMSO group, there was no statistically significant difference for the BMS754807 group) indicate significant differences among time points (P < 0.05, Newman-Keuls multiple comparisons test). For each time point, differences between DMSO and BMS754807 treated fish were analyzed by Student’s *t*-test (*p < 0.05; **p < 0.01; ***p <0.001). To confirm our findings, α-actin-EGFP were treated with the unrelated NVP-AEW541 Igf1R inhibitor. At 4 dpi the regenerating muscle was measured as before showing similar results (D); values are averages ± SD (Student's *t*-test, **p < 0.01, n = 5). To knock down Igf1r, lissamine-tagged MOs (red) against both Igf1r paralogs (a and b) were microinjected into α-actin-EGFP (green) fish muscles prior myectomy. MOs were detected through the whole regenerating muscle, including the distal ends (arrowhead). Control MO (up) and *Igfra/b* MO (down) injected fish are shown (E). The length of the regenerating muscle was measured as described (F); values are averages ± SD (Student’s *t*-test, *p < 0.05, n = 10). Diagram of a craniectomized zebrafish head (G); muscles visualized by this technique are shown, and LR muscles are highlighted in green. Green and red boxes show approximately the picture used for regeneration or LysoTracker Red and GFP-Lc3 (shown in Fig 2) assessment, respectively. The white arrows mark the growing end of the regenerating muscle. P, pituitary; e, eye. Gray box in panels C, D and F represent the 50% muscle length as baseline following myectomy.

For the time course experiment (Fig 1C), differences among time points for each fish group were analyzed by one-way analysis of variance (ANOVA) while differences between fish groups at each time point were analyzed by Student’s *t*-test. Thus, when more than 2 groups were compared, ANOVA (p < 0.05) followed by Newman-Keuls multiple comparisons test (p < 0.05) was performed. Comparisons between 2 groups were analyzed by Student’s *t*-test (*p < 0.05; **p < 0.01; ***p < 0.001). All tests were performed using the statistical software Prism 7.0a (GraphPad, LaJolla, CA, USA) for Mac OS X (Apple, Cupertino, CA, USA).

## Results

### Inhibition of Igf signaling impairs adult zebrafish muscle regeneration

Based on its known roles in muscle biology [12–16, 37–39], including EOMs [21–26], as well as in zebrafish heart and fin regeneration [10, 11], we decided to test the role of Igf signaling in the EOM regeneration of adult zebrafish. We used the drug BMS754807, selective inhibitor of Igf1r kinase [27] that has been shown to be effective in zebrafish [40–42]. Transgenic α-actin-EGFP fish were myectomized and daily treated with fresh BMS754807 for 3 days (Fig 1A and 1B). The measured length of the regenerating muscle was significantly lower in BMS754807 treated fish compared with control (Fig 1C—a gray area represents the residual muscle left after surgery [48.42 ± 4.9%, average ± S.D.] in the figures), revealing a role for Igf in EOM regeneration. To differentiate between a transient and a persistent action of Igf, we extended the drug treatments to 5 and 7 dpi. The length of the regenerating muscle was always lower in the treated fish (Fig 1C). The control fish (DMSO treated) showed a significant increase in muscle length over time while the treated fish did not show any significant muscle growth during the experimental period (Fig 1C), suggesting a key role of Igf in EOM regeneration. To confirm the specificity of the drug treatment, we utilized NVP-AEW541, an optimized Igf1r kinase inhibitor non-related to BMS754807 [28]. The lateral rectus of transgenic α-actin-EGFP fish was injured as before and fish were treated with NVP-AEW541 for 4 days. The regenerating muscle length was significantly lower in the NVP-AEW541 treated fish (Fig 1D), validating our previous results.

The key elements of Igf signaling are well conserved throughout evolution. However, due to the genome duplication in teleosts [43], two Igf1 receptor genes (*igf1ra* and *igf1rb*) have been described in zebrafish [44, 45]. To further test whether Igf signaling plays a role in muscle regeneration, Igf1ra/b expression was knocked down using previously described translation-blocking antisense MOs [32, 33], which were delivered via microinjection followed by electroporation prior to myectomy. LR muscle regeneration at 7 dpi was measured as before (Fig 1E) showing that knockdown of Igf1r expression effectively reduced LR muscle regeneration (Fig 1F).

### Myocyte dedifferentiation is not regulated by Igf signaling

**Fig 2:**
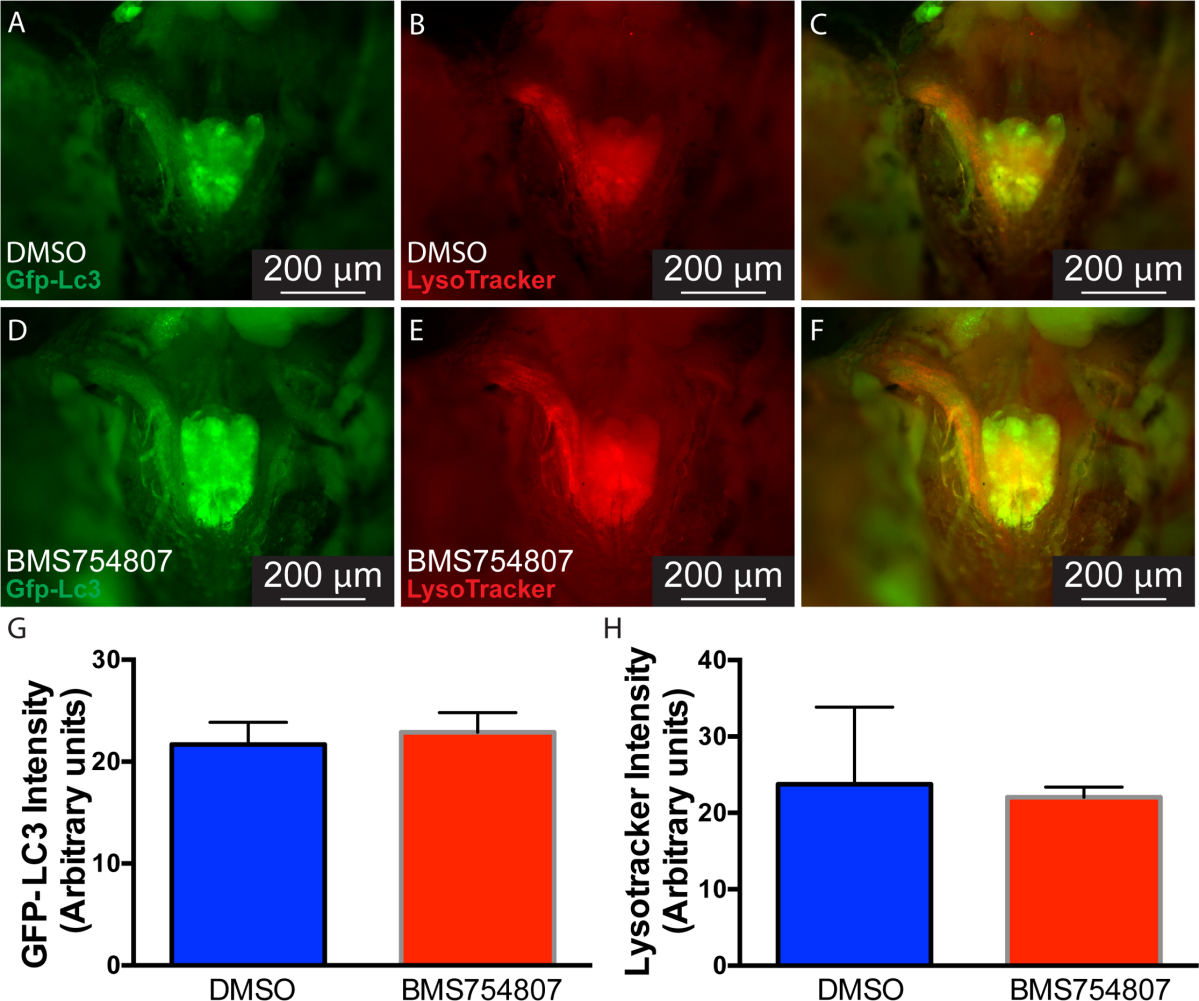
Inhibition of Igf signaling does not affect autophagy activation in the regenerating muscle. GFP-LC3 (A and D) fish were myectomyzed and LysoTracker Red (B and E) was used to label autophagy in the regenerating LR in fish treated with DMSO (A-C) or BMS754807 (D-F). C and F show the merging of A-B and D-E, respectively. GFP-LC3 (G) and LysoTracker (H) fluorescence intensity of the regenerating muscle were measured and no statistically significant difference between DMSO and BMS754807 treated fish was found. Values represent average ± SD (Student’s *t*-test, significance set at P < 0.05, n = 5). P, pituitary.

As previously determined, zebrafish EOMs activate autophagy after myectomy to degrade the sarcomeric contractile machinery and recycle specific myonuclei [7]. Map1lc3a/b is a protein ubiquitously distributed in the cytoplasm. After autophagy induction, the soluble form (Lc3-I) is conjugated to a form (Lc3-II) that localizes to the autophagosome membrane and accumulates within it [34]. LysoTracker Red, a vital dye that accumulates in autolysosomes and other acidic compartments, was used to stain transgenic GFP-Lc3 fish that were myectomyzed and treated with BMS754807 as before. Both GFP-Lc3 (Fig 2A and 2D) and LysoTracker Red (Fig 2B and 2E) clearly accumulated at 18 hpi in the injured muscle of BMS754807 treated and control (DMSO) fish, indicating autophagy activation. When GFP-Lc3 (Fig 2G) and LysoTracker Red (Fig 2H) fluorescence intensity was measured, no statistically significant differences were found indicating that, in EOM regeneration, autophagy activation is not under control of Igf signaling.

**Fig 3:**
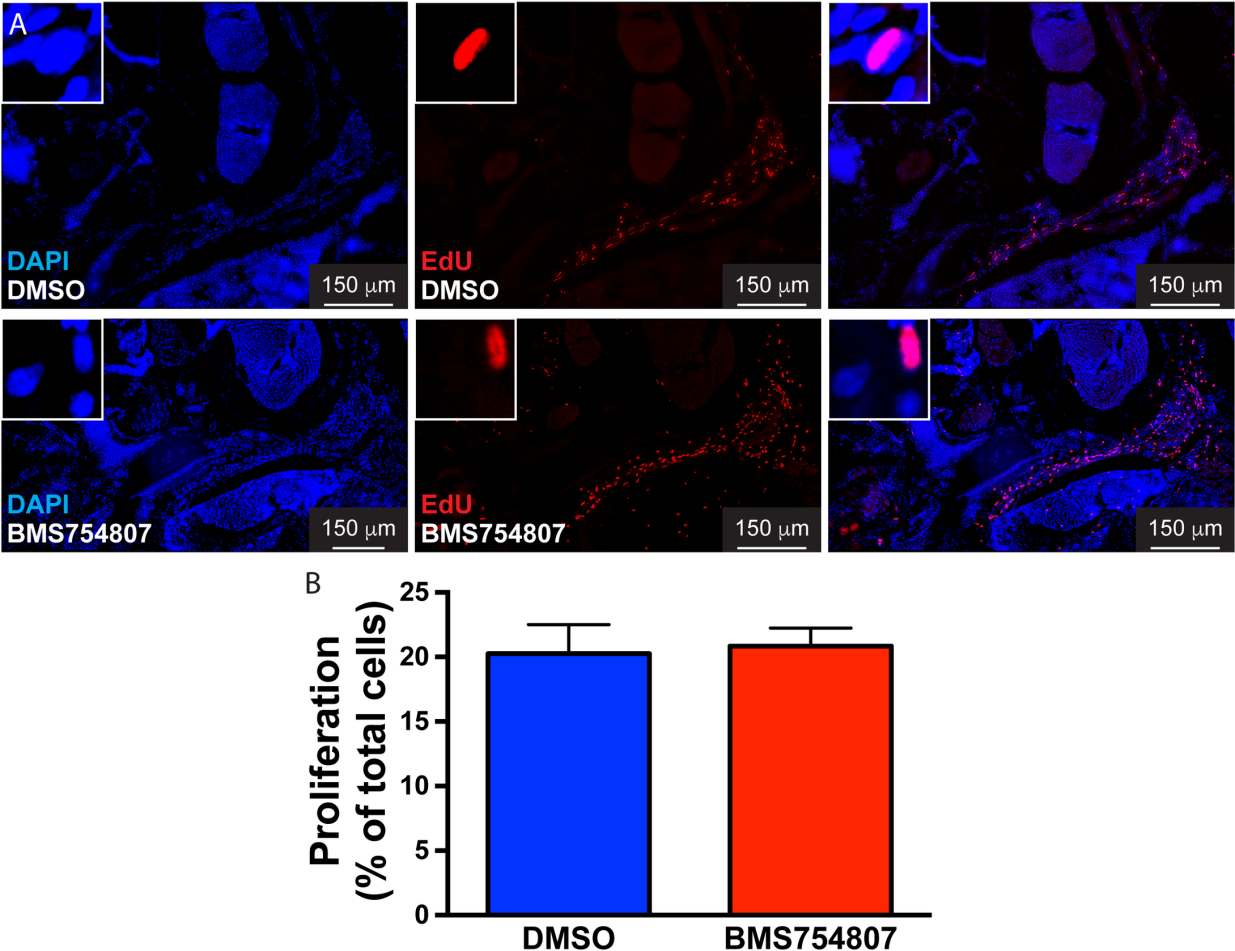
Inhibition of Igf signaling does not modify cell proliferation in the regenerating muscle. The role of Fgf in cell proliferation was assessed at 27 hours post-injury treating fish with DMSO or BMS754807 (A). DAPI, blue; EdU, red. Cell proliferation in the injured muscles from DMSO or BMS754807 treated fish was not statistically different (B). Values represent average ± SEM (Student’s *t*-test, significance set at P < 0.05, n = 4).

Myocyte dedifferentiation to myoblasts is required for the regeneration of adult zebrafish extraocular muscles. Myocyte dedifferentiation results in cell cycle reentry leading to a proliferative burst at 24–48 hpi. Then a gradual decline in proliferation occurs, followed by myoblast migration and myocyte re-differentiation [6]. Igf signaling could be important either in the early dedifferentiation steps leading to proliferation, and/or in the subsequent steps of migration and re-differentiation. In order to assess the role of Igf signaling in dedifferentiation, we used proliferation as readout. To label S-phase, zebrafish were treated with EdU at 23 hpi. EdU incorporation into dedifferentiated myocytes was assessed at 27 hpi. The percentage in the regenerating muscle of EdU positive cells was not affected by the treatment with the Igf1r inhibitor BMS754807 (Fig 3A and 3B). When combined, these observations suggest that myocyte reprogramming and dedifferentiation after injury is not regulated by Igf signaling.

### Inhibition of Igf signaling interferes with muscle re-differentiation

**Fig 4:**
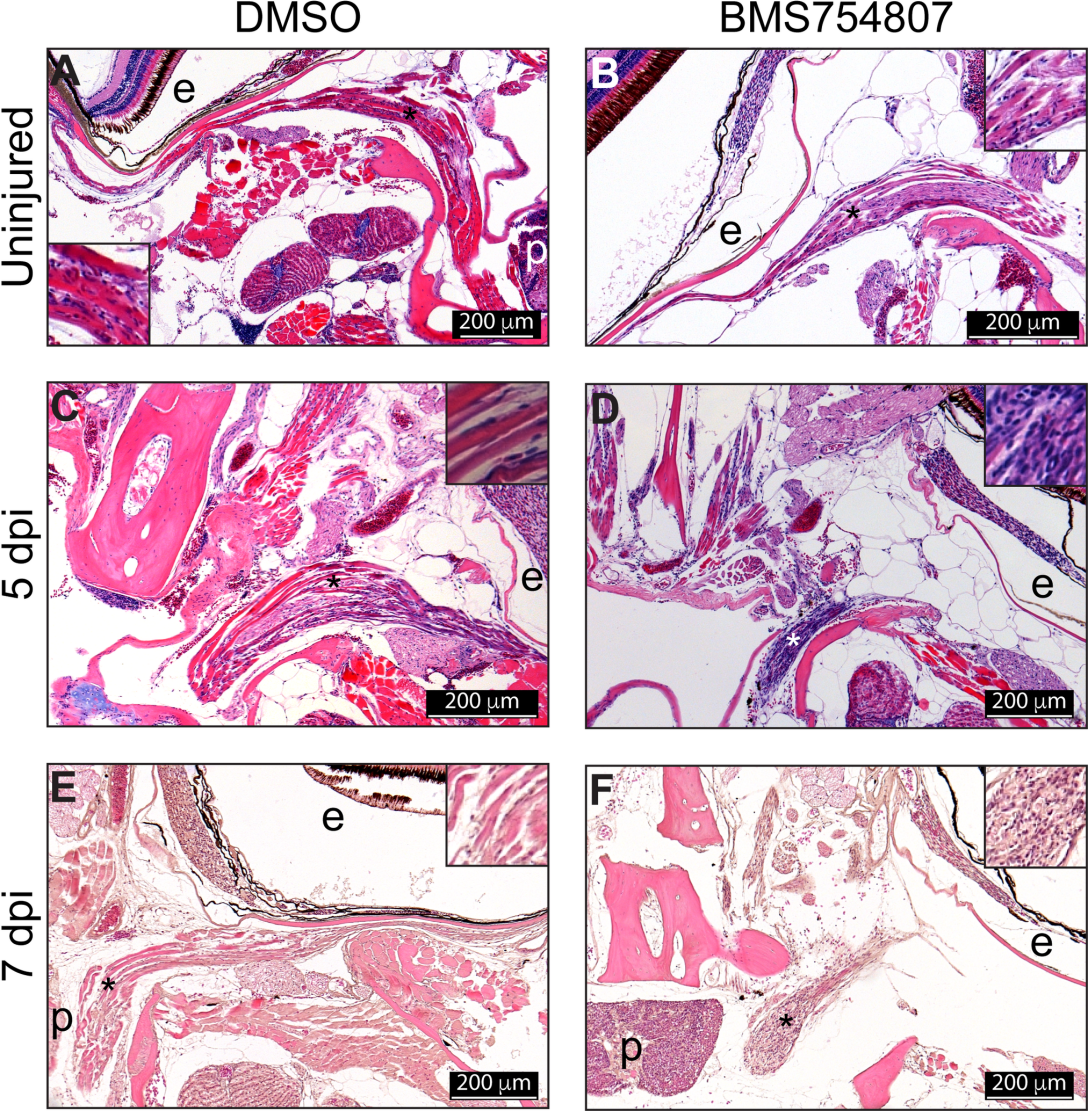
Histological analysis of the regenerating muscle. Paraffin sections (5 μm) H&E staining of regenerating muscles from DMSO (A, C) and BMS754807 (B, D) treated fish. Sections of regenerating muscle at 5 (A, B) and 7 dpi (C, D). The asterisk marks the approximate position of the inset. Images are representative examples from 5 fish analyzed per treatment and time point. P, pituitary; e, eye. The S1 Fig shows a zebrafish coronal section diagram as a reference for the position of the pictures shown in this figure.

In the absence of data supporting a contribution of Igf signaling to the dedifferentiation of injured myocytes during EOM regeneration, we performed a histological analysis of the regenerating EOMs (Fig 4). Hematoxylin and eosin (H&E) staining was performed at 5 and 7 dpi in fish treated with the Igf1r inhibitor BMS754807. The analysis of control fish (DMSO treated fish) showed a progressive re-differentiation of the muscle after injury displaying the typical muscle fiber structure (Fig 4A and 4C). Contrarily, the regenerating muscle of BMS754807 treated fish did not show differentiated muscle fibers and there was a general hypercellularity (Fig 4B and 4D). Thus, the histological analysis of the regenerating EOMs suggests a role of IGF in re-differentiating injured muscles.

**Fig 5:**
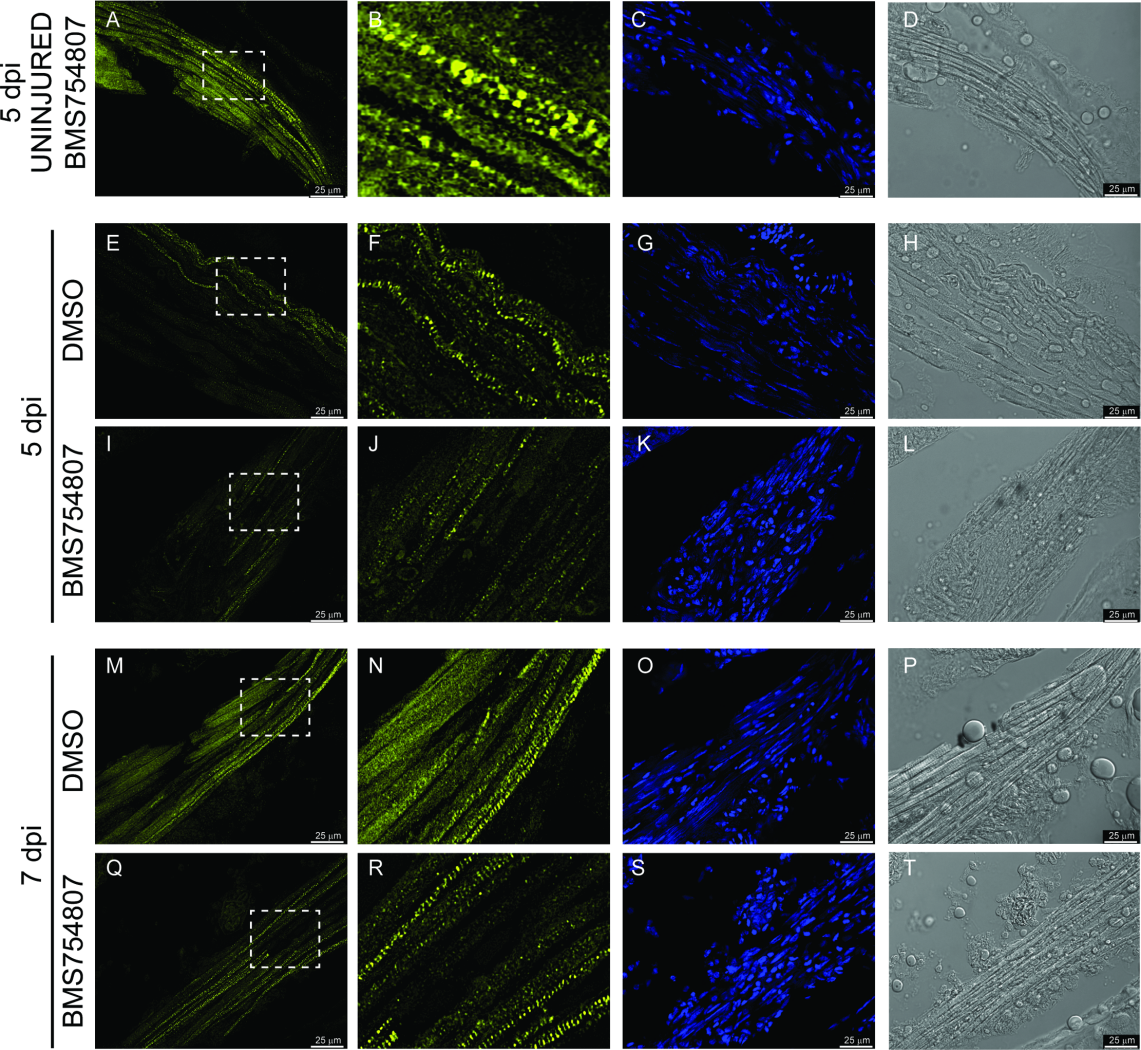
Myosin staining of the regenerating muscle. The effect of Igf signaling inhibition on EOM regeneration was analyzed using myosin expression as a marker of muscle differentiation. Uninjured EOMs of BMS754807 treated fish showed high levels of myosin staining (A, B), that were not different of those of a DMSO treated control fish. Myosin staining (yellow) of DMSO control fish at 5 dpi (E, F) and 7 dpi (M, N) reveal higher protein levels than the myosin staining of BMS754807 treated fish at 5 dpi (I, J) and 7 dpi (Q, R). The dashed box shows the approximate position of the magnification shown in B, F, J, N and R. DAPI staining of the corresponding myosin staining picture (C, G, K, O and S) and DIC images (D, H, L, P and T) are also shown. DAPI staining shows hypercellularity in BMS754807 (K, S compared to C, G, O) and typical elongated muscle nuclei in DMSO 7 dpi (O) and Uninjured EOMs (C). Pictures are representative examples of 7 fish per group, time and treatment.

To further test for the impairment of re-differentiation in regenerating EOMs, zebrafish were treated with BMS754807 as before and stained for heavy chain myosin (MHC, Fig 5), a marker of differentiated muscle fibers [46, 47]. The injured muscle of DMSO treated control fish shows an increase in MHC staining from 5 dpi (Fig 5E and 5F) to 7dpi (Fig 5M and 5N). In fact, by 7 dpi, the MHC staining of a DMSO control fish (Fig 5Q and 5R) injured muscle is very similar to that of an uninjured muscle (Fig 5A and 5B). Conversely, the MHC staining of injured muscles of BMS754807 treated fish was clearly less intense both at 5 dpi (Fig 5I and 5J) and 7dpi (Fig 5Q and 5R) dpi, when compared to that of DMSO control fish, indicating a defect, or at least a delay, in muscle differentiation when Igf signaling was inhibited. Moreover, nuclear DAPI staining supports our previous observations of hypercellularity (Fig 5K and 5S compared to Fig 5G and 5O) and reveals the presence of elongated nuclei at 7 dpi, typical of muscle fibers[48, 49], only in DMSO treated fish (Fig 5O) but not in BMS754807 treated fish (Fig 5S). All combined, these results indicate that, when Igf signaling is inhibited, regenerating EOMs are not able to properly re-differentiate muscle fibers from reprogrammed and dedifferentiated myoblasts.

### Role of Akt in zebrafish muscle regeneration

**Fig 6:**
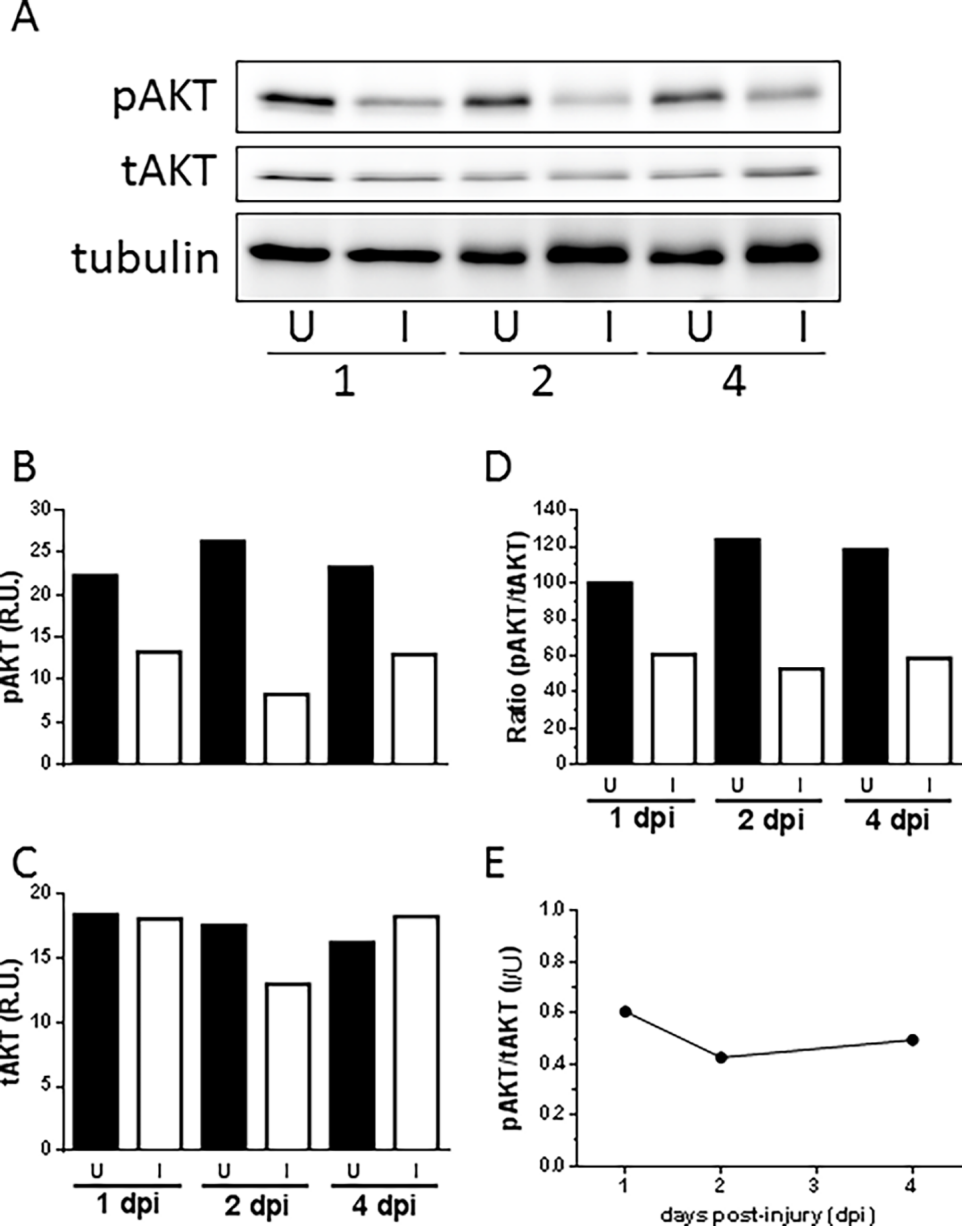
Role of Akt in the regenerating muscle. The activation of Akt in injured muscles (non-BMS754807 treated fish) was assessed by western blot in a time course experiment (A). Immunoblotting was performed with anti-phosphorylated Akt antibody. Total amounts of Akt were monitored by reprobing membranes with anti-Akt antibody. Note that phosphorylated Akt (pAkt) was rapidly and persistently reduced in the injured muscle. Tubulin was used as a loading control. The densitometric quantification of the Akt bands is shown (B, C). The intensity of pAkt (B) and tAkt bands (C) was normalized to the tubulin content. The ratio between pAkt and tAkt was used to represent the fraction of active Akt (D). For comparative purposes, the pAkt/tAkt ratio of the injured muscle was divided by the pAkt/tAkt ratio of the uninjured muscle at each time point (E). U, uninjured muscle; I, injured muscle; R.U., relative units.

The kinase Akt, also known as PKB (protein kinase B), is considered the main mediator of IGF actions in muscle development and regeneration. Upon ligand binding, Igf1r phosphorylates insulin receptor substrate (IRS). Phosphorylated IRS activates phosphatidylinositol-3-kinase (PI3K) which generates phosphoinositide-3,4,5-triphosphate (PIP3). PIP3 acts as a docking site for two kinases, phosphoinositide-dependent kinase 1 (PDK1) and Akt. The phosphorylation of Akt by PDK1 leads to its activation [50]. To determine the role of this pathway in EOM regeneration, the time course of Akt activation in the injured muscle was examined by western blot (Fig 6). Both forms of Akt, phosphorylated (pAkt) and total (tAkt), could be detected at 1, 2 and 4 dpi in uninjured muscles by western blot (Fig 6A). Following myectomy, phosphorylated and total Akt were still detectable by western blot in the injured muscle at 1, 2 and 4 dpi (Fig 6A). However, the levels of phosphorylated (active) Akt were lower than in uninjured muscles at 1 and 2 dpi (Fig 6A), as revealed by the band intensity quantification of phosphorylated (active, Fig 6B) and total Akt (Fig 5C); and the ratio between them (Fig 5D). The levels of phosphorylated (active) Akt remained low in injured muscles even 4 days after myectomy (Fig 5A, 5B and 5D), when the injured muscle was already regenerating (Fig 1). These results suggest that the actions of Igf in zebrafish muscle regeneration are not mediated by Akt.

## Discussion

This work focuses on the regulatory role of Igf in zebrafish extraocular muscle (EOM) regeneration. In adult zebrafish, EOM regeneration begins with myocyte dedifferentiation followed by proliferation and migration of reprogrammed myoblasts, and eventually redifferentiation into myocytes that fuse to form myofibers [6]. As expected, Igf signaling inhibition impaired muscle regeneration (Fig 1), supporting the described role of Igf in promoting zebrafish tissue regeneration [10, 11], EOM plasticity and force regulation [23–25] and muscle repair more generally [12–16, 37–39].

We identified autophagy as an essential early step of EOM regeneration[7]. Given the role of Igf signaling promoting [18] or inhibiting [17] autophagy, we tested whether Igf inhibition altered autophagy in EOM regeneration. Surprisingly, using both LysoTracker Red staining and GFP-Lc3 accumulation, we found that autophagy activation is Igf-independent (Fig 2). The most important early step of regeneration is cell cycle reentry by dedifferentiated myoblasts, leading to a proliferative burst [6]. Our results reveal that Igf signaling is dispensable for this early step as well (Fig 3). A histologic analysis of injured EOMs in which Igf signaling had been inhibited revealed hypercellularity and relative absence of differentiated muscle fibers even days following injury (Fig 4), suggesting defects in the late steps of EOM regeneration. Since Igf is known to play a role in myoblast terminal differentiation [51–53], we tested the hypothesis that Igf signaling is necessary for redifferentiation of proliferating myoblasts. Staining for myosin, a classical marker of muscle differentiation [46, 47], we discovered that indeed myocyte redifferentiation was inhibited or severely delayed, providing a mechanistic explanation for the observed delay in EOM regeneration.

Since Akt is considered a primary mediator of Igf signaling in myogenesis [50], we tested for the presence of phospho-Akt in injured EOMs (untreated). Interestingly, phospho-Akt levels decreased after injury (Fig 6), making it extremely unlikely that Akt mediates Igf actions in adult de novo EOM regeneration. Interestingly, in old-age transgenic mice expressing Igf-1, muscle reparative capacities were similar to those of young mice [54] but with no effect on Akt activation [55]. Therefore, Igf signaling in the context of adult skeletal muscle regeneration must function through a different pathway in both mice and zebrafish. The mitogen-activated protein kinase/extracellular signal-regulated receptor kinase (MAPK/ERK) signaling pathway was reported as an alternative mediator of Igf actions in rat muscle [56–58]. We had previously described that Erk2 was selectively activated during the early steps of zebrafish EOM regeneration [8], but the data reported in the present manuscript reveal a late role for Igf signaling. Therefore, it is unlikely that the Erk pathway mediates Igf actions in EOM regeneration. These results suggest the existence of a yet-to-be-identified alternate pathway for Igf signaling in adult muscle repair and regeneration.

In conclusion, using an *in vivo* model of zebrafish EOMs, we report that Igf signaling is important for the redifferentiating steps of muscle regeneration, and that this role may be shared with muscle reparative pathways in adult mammals. Despite the obvious differences among the models, studying adult zebrafish EOM regeneration may facilitate a mechanistic understanding of a skeletal muscle’s response to injury and highlight opportunities for novel therapeutic development. Furthermore, understanding the biological mechanisms underlying early de-differentiation vs. late re-differentiation of injured adult tissues enhance our understanding of regenerative pathways in general, irrespective of tissue type.

## Supporting information

S1 Fig Diagrams of the pictures shown in Fig 4. Diagrams of coronal zebrafish head sections from DMSO (A, C) and BMS754807 (B, D) treated fish. Sections of regenerating muscle at 5 (A, B) and 7 dpi (C, D). Dashed box shows the approximate location of the picture shown in Fig 4. For reference, the asterisk is approximately located in the same position than in Fig 4. Approximate position of the sectioning plane (G).
(TIF)
