# Supplementary figures and images for: Extraocular muscle regeneration in zebrafish requires late signals from Insulin-like growth factors
Source: PLoS One. 2018 Feb 7;13(2):e0192214. doi: 10.1371/journal.pone.0192214 (PMC5802911; doi:10.1371/journal.pone.0192214)

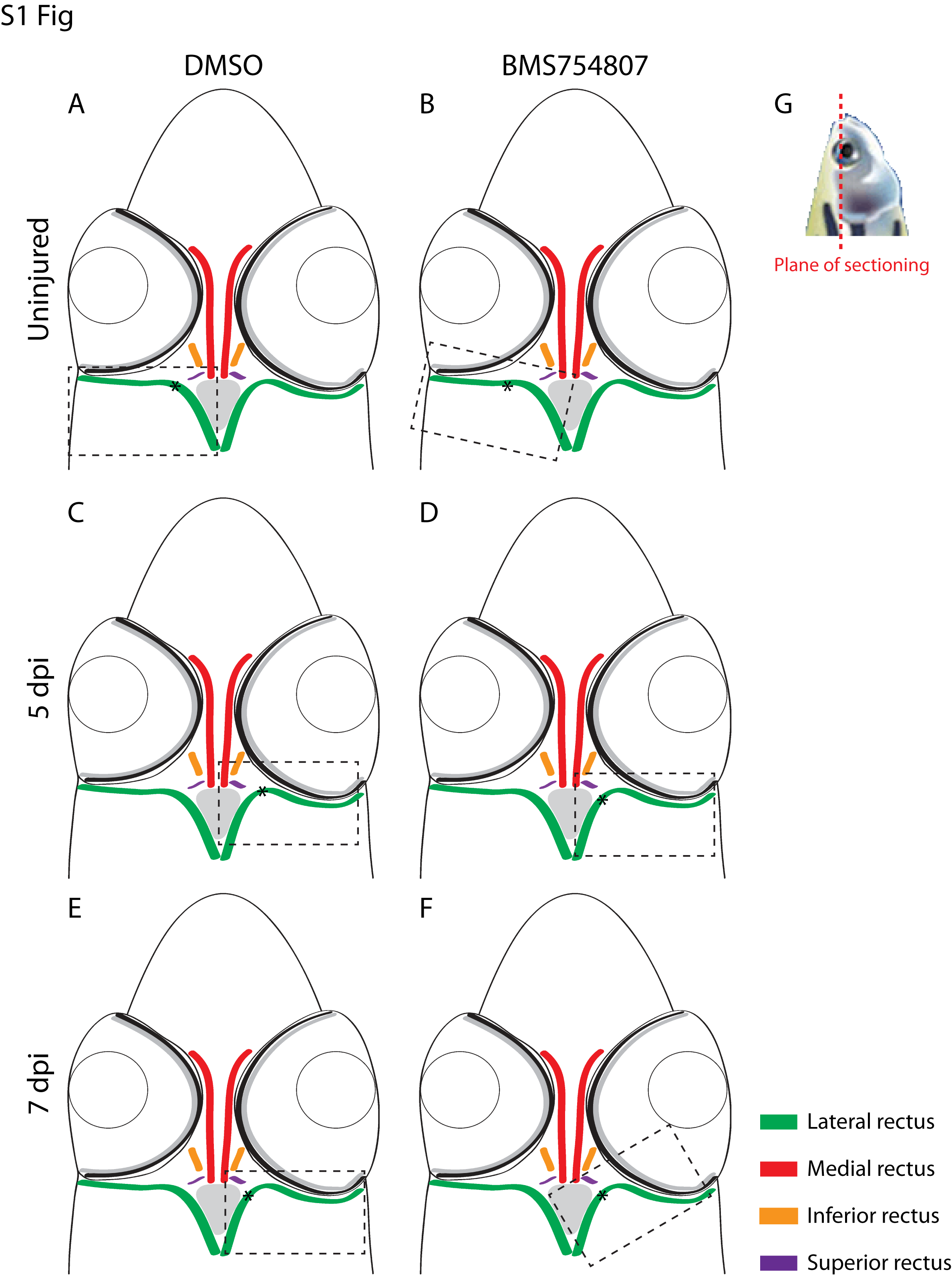

Supplement: S1 Fig — Diagrams of coronal zebrafish head sections from DMSO (A, C) and BMS754807 (B, D) treated fish. Sections of regenerating muscle at 5 (A, B) and 7 dpi (C, D). Dashed box shows the approximate location of the picture shown in Fig 4. For reference, the asterisk is approximately located in the same position than in Fig 4. Approximate position of the sectioning plane (G). (TIF) [file pone.0192214.s001.tif]
